# Predicting Social Determinants of Health in Patient Navigation: Case Study

**DOI:** 10.2196/42683

**Published:** 2023-03-28

**Authors:** Francisco Iacobelli, Anna Yang, Laura Tom, Ivy S Leung, John Crissman, Rufino Salgado, Melissa Simon

**Affiliations:** 1 Department of Computer Science Northeastern Illinois University Chicago, IL United States; 2 Center for Advancing Safety of Machine Intelligence Northwestern University Evanston, IL United States; 3 Center for Health Equity Transformation Feinberg School of Medicine Chicago Northwestern University Chicago, IL United States; 4 Department of Obstetrics and Gynecology Feinberg School of Medicine Chicago Northwestern University Chicago, IL United States; 5 Robert H. Lurie Comprehensive Cancer Center Feinberg School of Medicine Chicago Northwestern University Chicago, IL United States

**Keywords:** patient navigation, machine learning, social determinants of health, health care disparities, health equity, case study

## Abstract

**Background:**

Patient navigation (PN) programs have demonstrated efficacy in improving health outcomes for marginalized populations across a range of clinical contexts by addressing barriers to health care, including social determinants of health (SDoHs). However, it can be challenging for navigators to identify SDoHs by asking patients directly because of many factors, including patients’ reluctance to disclose information, communication barriers, and the variable resources and experience levels of patient navigators. Navigators could benefit from strategies that augment their ability to gather SDoH data. Machine learning can be leveraged as one of these strategies to identify SDoH-related barriers. This could further improve health outcomes, particularly in underserved populations.

**Objective:**

In this formative study, we explored novel machine learning–based approaches to predict SDoHs in 2 Chicago area PN studies. In the first approach, we applied machine learning to data that include comments and interaction details between patients and navigators, whereas the second approach augmented patients’ demographic information. This paper presents the results of these experiments and provides recommendations for data collection and the application of machine learning techniques more generally to the problem of predicting SDoHs.

**Methods:**

We conducted 2 experiments to explore the feasibility of using machine learning to predict patients’ SDoHs using data collected from PN research. The machine learning algorithms were trained on data collected from 2 Chicago area PN studies. In the first experiment, we compared several machine learning algorithms (logistic regression, random forest, support vector machine, artificial neural network, and Gaussian naive Bayes) to predict SDoHs from both patient demographics and navigator’s encounter data over time. In the second experiment, we used multiclass classification with augmented information, such as transportation time to a hospital, to predict multiple SDoHs for each patient.

**Results:**

In the first experiment, the random forest classifier achieved the highest accuracy among the classifiers tested. The overall accuracy to predict SDoHs was 71.3%. In the second experiment, multiclass classification effectively predicted a few patients’ SDoHs based purely on demographic and augmented data. The best accuracy of these predictions overall was 73%. However, both experiments yielded high variability in individual SDoH predictions and correlations that become salient among SDoHs.

**Conclusions:**

To our knowledge, this study is the first approach to applying PN encounter data and multiclass learning algorithms to predict SDoHs. The experiments discussed yielded valuable lessons, including the awareness of model limitations and bias, planning for standardization of data sources and measurement, and the need to identify and anticipate the intersectionality and clustering of SDoHs. Although our focus was on predicting patients’ SDoHs, machine learning can have a broad range of applications in the field of PN, from tailoring intervention delivery (eg, supporting PN decision-making) to informing resource allocation for measurement, and PN supervision.

## Introduction

Patient navigation (PN) programs have demonstrated efficacy in improving health outcomes in marginalized populations across a range of clinical contexts. Initially developed in 1990 to address breast cancer disparities [1], PN has since been tested in other medical fields, demonstrating improved outcomes for other cancers [2], dementia [3], depression [4], sickle cell disease [5], and complex multimorbidity in children [6] and older adult populations [7], among other conditions. PN programs in the United States have typically focused on serving marginalized populations who experience health care inequities, including low-income individuals [8], racial and ethnic minority groups, immigrants and refugees, inner-city residents [9-12], and rural residents [13,14]. Across the board, a growing body of literature suggests that navigation is associated with increased preventive services use (such as cancer screenings) and follow-up, earlier detection of health abnormalities, earlier or lower clinical stage of presentation, higher patient satisfaction, improved outcomes during survivorship, and considerably reduced health care disparities [15].

The widespread success of PN programs comes largely from their effectiveness in alleviating patient barriers to accessing health care and improving the timeliness of diagnosis, follow-up, and treatment [16]. Research has identified a myriad of barriers addressed by PN, including being uninsured or underinsured, financial barriers, language discordance, housing issues, transportation difficulties, and fear or mistrust [17]. Often, patient navigators were found to be crucial in helping individuals navigate complex bureaucracy within local health care systems [18]. Some barriers that navigators address are population-specific and other disease specific, but a common underlying thread is social determinants of health (SDoHs), defined by the Centers for Disease Control and Prevention as the “conditions in the places where people live, learn, work, and play that affect a wide range of health and quality-of-life risks and outcomes” [19]. SDoHs are a well-recognized driver of diverse health inequities across populations [20].

In ideal circumstances, SDoH information for patients can be collected directly by health care providers and used to optimize patient-centered care. However, it can be challenging for providers to identify SDoH-related barriers experienced by patients owing to a lack of time to ask [21], workflow integration difficulties [22], prevalent data gaps [23], the lack of standardized screening tools [24], and the lack of providers competent in identifying SDoHs or those who come from low-income backgrounds [25]. In recent years, several tools have emerged to help clinical care providers identify patients’ SDoHs, including the Protocol for Responding to and Assessing Patients’ Assets, Risks, and Experiences; the Accountable Health Communities Health-Related Social Needs Screening Tool; and the International Classification of Diseases, Tenth Revision codes in categories Z55 to Z65 (Z codes) [26-28]. These strategies for identifying SDoHs have several limitations, however, including ambiguous definitions, inconsistent thresholds in clinical settings, the lack of structural incentives for providers to screen and enter data into electronic health records, time and labor costs of training staff to adopt screening tools, and the limited ability of medical sites to address identified barriers [22,24,29,30]. Patient navigators experience many of these challenges.

Navigators who serve the function of addressing patient barriers to care typically identify barriers (including SDoHs) and risks for their patients through assessments and ongoing interactions, as navigators follow patients through a particular care continuum. Depending on patient needs at each point in their care, navigators may assist patients, for example, by scheduling appointments, coordinating referrals, making social service arrangements, providing health education, facilitating patient-provider communication, providing psychosocial support, and applying for health insurance [17]. However, for each patient, it takes time for navigators to build sufficient patient rapport to solicit a full picture of a patient’s SDoHs, as well as the fine-tuning of cultural competency and communication skills [31]. Many patients who navigators serve experience a multiplicity of barriers that intensifies the challenges that navigators face in identifying SDoH barriers. Indeed, many patients’ SDoH-related barriers require more than one navigation encounter to uncover, owing to a myriad of factors, including patients’ reluctance to disclose information, communication barriers, and the variable experience level of patient navigators. Patient navigators could benefit from strategies that augment their current abilities to gather SDoH data to efficiently identify and resolve social services and other SDoHs needs in a timely manner.

In exploring strategies to augment the work of patient navigators in identifying and mitigating SDoH-related patient barriers, we turn to machine learning—the use of computational techniques to detect patterns in data and predict outcomes—to predict the SDoHs to create patient profiles that potentially enhance and optimize the effectiveness of PN in improving health outcomes for diverse patient populations. For example, by creating a predicted profile of SDoHs for a patient, the navigator can bolster information from existing SDoH assessments, find some guidance as to what aspects of SDoHs screening to pay more attention to, and conduct interactions to screen for specific SDoHs that may not be self-evident. This helps both less experienced navigators detect SDoHs, as well as optimize the time of underresourced navigators.

There has been a growing interest in the medical community in the promise of machine learning and its potential contributions to detecting and predicting SDoHs. For example, Kasthurirathne et al [32] used patient clinical data and community data representing SDoHs to predict the need for congruous social services; their results regarding sensitivity, specificity, and accuracy fell between 60% and 75%. Abarca-Alvarez et al [33] created a model to describe and predict social vulnerability based on the demographic and geographic characteristics obtained from census data. Researchers have also attempted to predict concrete outcomes using community, geographic, and social indicators, including stillbirth [34], uncontrolled type 2 diabetes [35], and BMI [36]. However, community data do not necessarily predict individual needs [37]. Most studies using machine learning have used data from textual surveys, and few have augmented these data with images, text, or sound [38]. However, we identified 3 gaps in the research on machine learning algorithms for identifying SDoHs automatically, especially in the PN context. First, although clinical notes with social workers’ notes have been used to predict some SDoHs [39], the combination of textual and demographic information has not been attempted in the classification of multiple SDoHs. Second, although community data have been used in previous studies, these usually involve an aggregate of geographic area data, not personalized community data (such as the proximity of each individual to the nearest hospital) [38]. Finally, patients often experience multiple SDoHs at a time, but existing research has only used single classification algorithms instead of multilabel classification, which can, in theory, detect richer and more accurate co-occurrences of patients’ SDoHs [38].

To address these gaps, we report the results and lessons learned in 2 experiments applying machine learning algorithms to PN data collected from 2 PN studies in the Chicago area. In the first experiment, we compared machine learning algorithms to predict SDoHs from both patient demographic data and navigators’ textual patient encounter notes to determine whether 1 or more algorithms are suitable for this task. In the second experiment, we used multilabel classification with personalized augmented information from the Google application programming interface (API) to predict SDoHs from an initial demographic profile. The reported case study and lessons learned can inform the use of machine learning for future PN programs and other initiatives that seek to identify and address SDoH barriers to care for marginalized populations.

## Methods

### Experiment 1: Using Patient Navigator Encounter Notes

The goal of this experiment was to compare machine learning algorithms to predict SDoHs and determine whether 1 or more algorithms are suitable for this task. In particular, we explored whether demographic information alone or together with patient navigator notes can help predict patients’ SDoHs.

#### Data Set

The data used in experiment 1 were sourced from the Chicago Chinatown PN Program, a research study evaluating the effectiveness of PN to enhance breast and cervical cancer screening and follow-up among women residing in Chicago’s Greater Chinatown area [40]. The data were obtained from patient navigators’ tracking logs of 330 patients enrolled and navigated in the study between July 2013 and November 2018. After each patient interaction, the navigator entered a record of the encounter into the REDCap (Vanderbilt University) database. The data contained demographics of patients (Table 1), as well as information from each encounter involving a navigator interacting with a patient or care provider on behalf of a patient. PN encounter event records included notes left by the patient navigator, all languages spoken by the patient, the preferred language spoken, time spent with the physician, action taken, the length of action taken, barriers and related SDoHs labeled by a patient navigator, and the medium in which the meeting was held (eg, in-person or phone call; Table 2). There were 22 SDoH categories identified (Textbox 1). Notably, not all patients had the same number of PN encounters.

Machine learning algorithms are based on instances (encounters or patients) with attributes (discrete or continuous variables associated with each instance; eg, language spoken at home or age). The probabilistic nature of these algorithms requires textual fields to be converted into numerical attributes. Otherwise, the text associated with an instance may be too unique to help generalize the machine learning algorithm in predicting new instances. For example, a note for 1 patient specifying “[navigator] recommended pt to see GYN doctor” and another for a different patient saying, “email [navigator] to give her an appointment with one of the gyne” are similar to that of a human. However, for a computer, these are 2 data points that are completely different. However, there are algorithms that allow us to determine which words are similar and cluster similar words into groups called topics. Thus, we converted textual notes into a vector of topics using latent Dirichlet allocation (LDA), a natural language processing algorithm to group similar terms into clusters (topics) that has been widely used in machine learning classification tasks in which textual information is used [41].

A total of 5 machine learning algorithms were used to predict SDoHs using only patient demographic data. These 5 algorithms were logistic regression, random forest, support vector machine, artificial neural network, and Gaussian naive Bayes. These algorithms were chosen because of (1) their diversity of approaches—regression, decision trees, algebraic, nonlinear, and baseline probabilistic; (2) their fair performance on standard classification tasks; and (3) their wide availability for use by non–machine learning specialists. These models were trained using a subset of the patients and tested on new patients the model has not seen. Experiments were also conducted using patient demographic data, along with patient navigator encounter data. Some of the navigation encounter data were nonnumeric. Categorical data were converted using 1-hot encoding, which converts each value of an attribute into a new binary attribute. Values were represented by 1 or 0 to indicate whether the data point had acquired this attribute or not, respectively.

These data presented the following idiosyncratic challenges in the context of training machine learning algorithms: (1) patients did not necessarily have the same number of navigation encounters; and (2) some navigation encounters included more than one SDoHs, some listed none, and some documented an already reported SDoHs. Consequently, we attempted to prepare the data in different ways, in consultation with the Chicago Chinatown PN Program study team.

Six different strategies were used to prepare the data for the classification algorithms. Strategies 1 to 4 considered patient demographic data and data from each navigation encounter. Strategies 5 and 6 only involved patient demographic data.

**Table 1:** Demographic attributes of each patient and how missing values were handled for each patient.

Attributes	Type	How missing values were handled
Age	Numeric	Average
Occupation	Categorical	Most common
Marital status	Categorical	Most common
Education level	Categorical	Most common
What year came to United States	Numeric	Average
English speaking level	Categorical	Most common
Where are you from?	Categorical	Most common
Zip code	Numeric	Most common
How many live in house	Numeric	Average
Born in United States?	Categorical (binary)	Most common
Household income (range)	Categorical	Most common

**Table 2:** Data recorded for each patient navigation encounter, and how we dealt with missing data in our set.

Attributes	Type	How missing values were handled
Preferred language	Categorical	Most common
All languages spoken	Categorical	Most common
Type of service	Categorical	Most common
Channel	Categorical	Most common
Length of action taken	Numeric	Average
Action taken	Categorical	Most common
Comments	Text (sentence or sentences)	Did not include this visit
SDoH^a^ (label for data point)	Categorical (this stayed categorical because it was the label for our data points)	Did not include this visit

^a^SDoH: social determinant of health.

**Social determinants of health categories in the patient navigation tracking log.**

1. Navigator barriers
2. Transportation
3. Housing
4. Social/practical support
5. Language/interpreter
6. Literacy
7. Childcare issues
8. Family/community issues
9. Distance from health care facility
10. Insurance/uninsured/underinsured
11. Financial problems
12. Work schedule conflicts
13. Communication concerns with medical personnel
14. Fear
15. Medical and mental health comorbidity
16. Patient disability
17. Out of town/country
18. Perceptions/beliefs about tests/treatment
19. System problems with scheduling care
20. Attitudes toward providers
21. Citizenship
22. Other (write-in)

#### Preparation Strategy 1: Each Patient Is a Data Point

**Figure 1:**
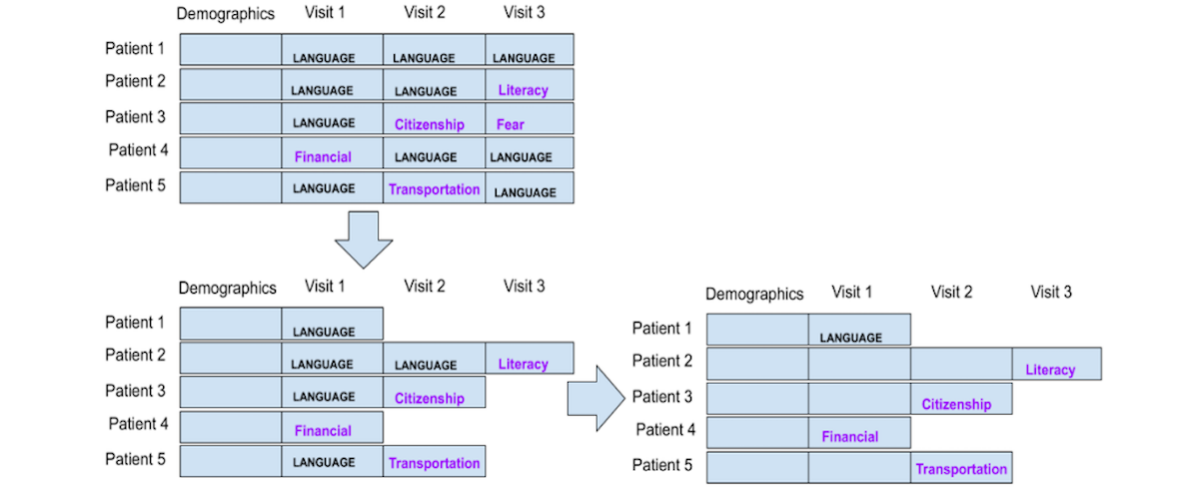
Strategy 1: each patient is a data point with 1 social determinant of health per encounter.

Strategy 1 considered each patient as a data point. Although navigators could report ≥1 of the 22 available SDoH categories for each navigation encounter, we used the overall frequency of occurrence to determine the most salient SDoHs in each encounter. Because the sample was biased toward Chinese speakers, if a patient had multiple navigation encounters in which the navigator determined the SDoH of “language/interpreter,” we aggregated the encounters leading up to the first occurrence of an encounter that was not “language/interpreter.” We labeled the data point or patient as the non–language or interpreter SDoH. If every encounter had an SDoH of “language/interpreter,” then we labeled that data point’s SDoH as “language/interpreter” and only considered the first navigation encounter for that patient because it only took 1 navigation encounter to determine the patient’s singular longitudinal SDoH. This reduced the weight algorithms assigned to that pervasive SDoH. With this strategy, we had 300 data points. Figure 1 illustrates this strategy.

#### Preparation Strategy 2: Each Patient Is a Data Point, Excluding the Language Barrier

**Figure 2:**
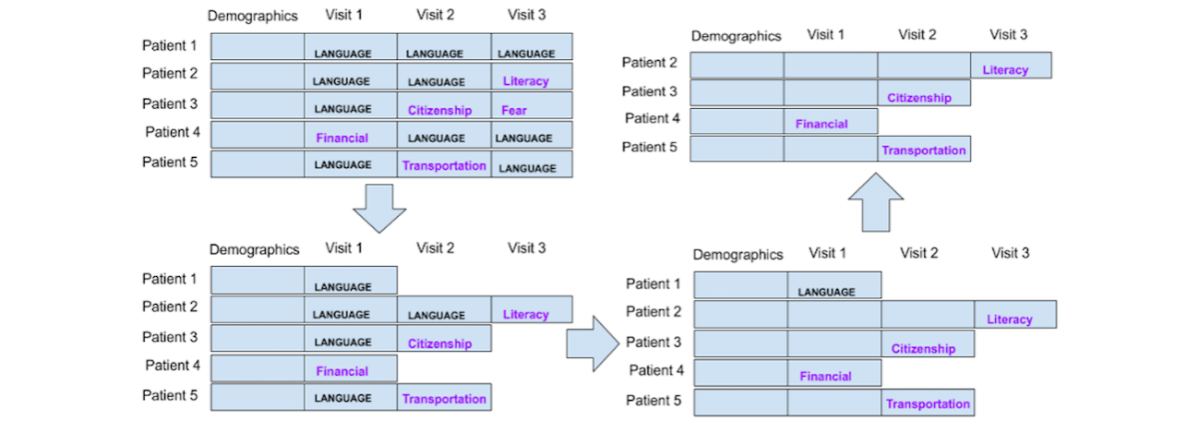
Strategy 2: each patient is an instance, but patients with only “language” as the social determinants of health barrier were excluded as data points.

This strategy is similar to strategy 1, except that once we have all the data points, we eliminated all the data tuples that only have “language/interpreter” as the SDoH label. Because most data points were labeled as “language/interpreter,” we wanted to see the effect of the attributes on the accuracy of predicting other SDoHs. Figure 2 illustrates this strategy.

#### Preparation Strategy 3: Each Encounter Is a Data Point

**Figure 3:**
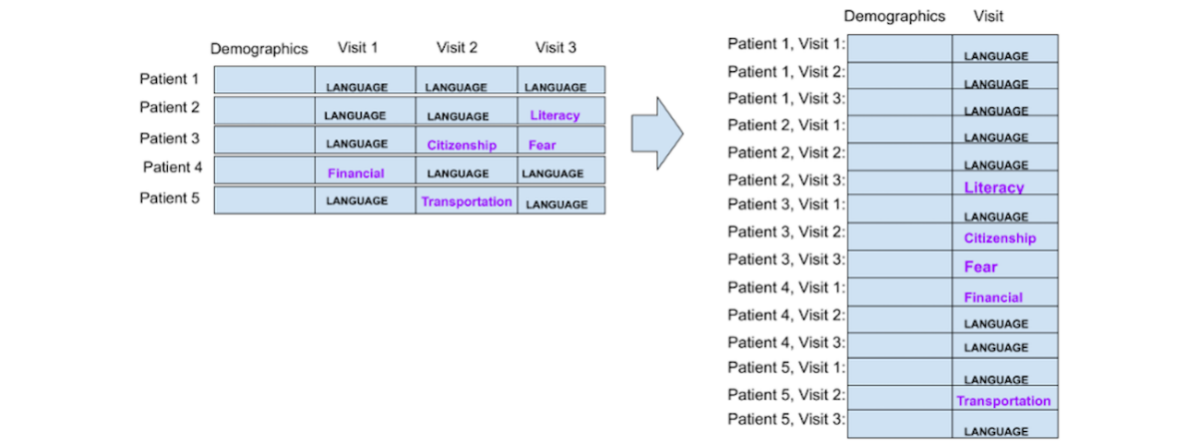
Strategy 3: each navigation encounter is a data point.

This strategy considers each navigation encounter, not each patient, as a data point, thereby generating a large number of data points. Patient demographic information was the same for many data points because multiple data points or encounters came from the same patient. With this strategy, we obtained >1400 data points. Figure 3 illustrates this strategy.

#### Preparation Strategy 4: Each Encounter Is a Data Point, Excluding the Language Barrier

**Figure 4:**
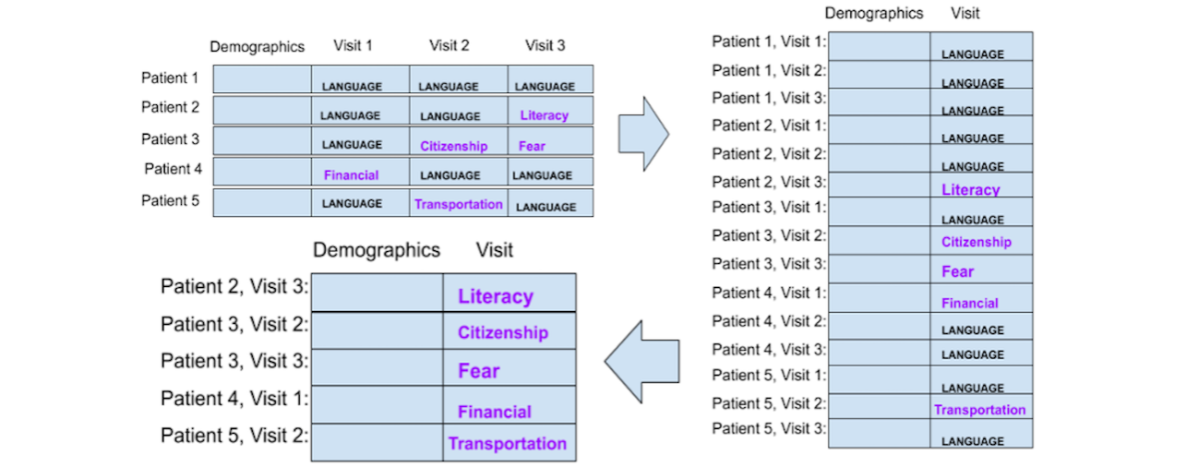
Strategy 4: each encounter is a data point, while “language” social determinants of health data points were excluded.

This strategy used the same technique as strategy 3 in considering each navigation encounter as a data point, but similar to strategy 2, we eliminated all the data points with an SDoH of “language/interpreter.” This strategy is illustrated in Figure 4.

#### Preparation Strategy 5: Each Patient Is a Data Point, and Only Demographic Data Are Included

**Figure 5:**
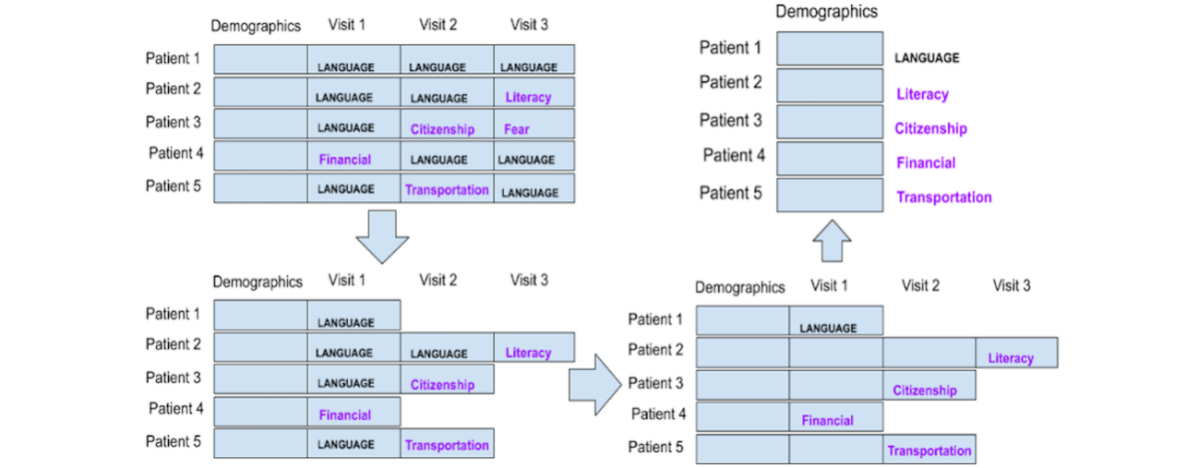
Strategy 5: each patient is a data point. After labeling the social determinants of health, navigation encounter information is excluded.

Strategy 5 considers each patient as a separate data point but only includes patient demographic data. Labels are obtained by following the same procedure as in strategy 1, except that the navigation encounter data are excluded. This is illustrated in Figure 5.

#### Preparation Strategy 6: Each Patient Is a Data Point, Only Demographic Data Are Included, and Language Barriers Are Excluded

**Figure 6:**
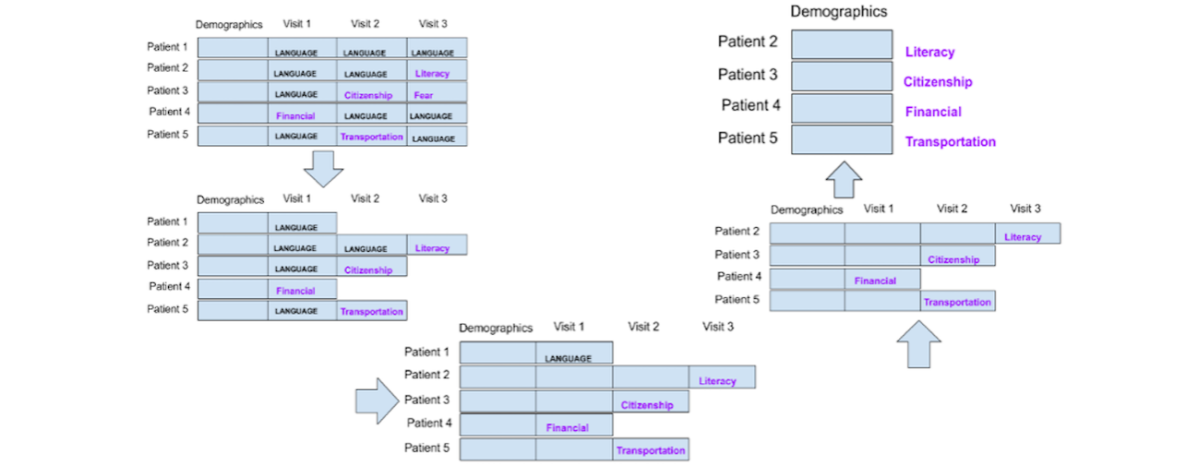
Strategy 6: each patient is an instance; labels of “language/interpreter” and encounter data were excluded.

Strategy 6 considers each patient as a separate data point, as in strategy 1, but we only considered the demographics data. As in strategy 2, patients with the label of “language/interpreter” were excluded. Figure 6 illustrates this strategy.

#### From Navigator Notes to Attributes

LDA was used to convert the comments of the navigator on each encounter (text) into meaningful numerical data. This is a generative statistical model used to capture textual documents into *k* latent topics (clusters of related words). Each document has a probability distribution of belonging to each of the *k* topics [41]. Therefore, a document becomes a series of *k* probability values. We used this approach to convert the text into *k* attributes. For each strategy that used navigation encounter data (strategies 1-4), we tested different numbers of topics or latent attributes (*k*), including *k*=5, *k*=10, *k*=15, *k*=20, *k*=25, and *k*=30 for the number of topics. Although methods to compute an optimum number of topics exist, these methods may lose important information in our data, which were in short notes form; therefore, we opted to test a variety of topic numbers instead.

Once all data were converted to numeric data and fully processed according to the 6 strategies, we tested 5 different machine learning algorithms to create models that can predict the SDoH. As mentioned earlier, LDA did not play a role in strategies 5 and 6 because encounter data were not used for these strategies. Thus, in total, we tested 130 different models (strategies 1-4 × 6 topic configurations × 5 algorithms + strategies 5-6 × 5 algorithms). These models were trained using a subset of the PN data and tested on PN data that the model had not yet seen (hereafter referred to as “new patients”). The 5 machine learning algorithms or classifiers that were used are as follows:

1. *Logistic regression* was the first machine learning algorithm used to predict SDoHs in new patients. Logistic regression is a statistical model that in its primary form uses logistic functions to model the behavior behind a binary class problem. In our data, we included 22 different classes of SDoHs. To make this a binary class problem, logistic regression considers 1 SDoH as 1 class (A) and the rest of the SDoHs as the other class (not A). The algorithm does this for every SDoH (class).
2. The second machine learning algorithm we used for prediction was a *random forest* classifier. A random forest classifier creates many decision trees during training. A test data point runs through each tree, and the prediction is determined by the label the most often determined by the decision trees. A decision tree is a rule-based classifier that tests attributes according to the entropy they contribute to the data. Attributes with less entropy are tested first.
3. The third machine learning algorithm is a *support vector machine*. Support vector machines take data in higher dimensions and separate them from the hyperplanes in a lesser dimension. New data points will be classified depending on where they lay in the N-dimensional space. This will depend on how the N-1 dimensional hyperplane separates from the N-dimensional space.
4. *Artificial neural networks* were used as the fourth machine learning algorithm. This algorithm is a function modeled after the biological neural networks in the human brain. The algorithm consists of initializing the weights and biases, forward propagation, calculated costs, backpropagation, and convergence to a local minimum. Each node in an artificial neural network is called an artificial neuron and is very similar to a biological neuron because it takes some input and sends out some output. Artificial neural networks take in and output only numbers.
5. The fifth algorithm we used was *Gaussian naive Bayes*, which is based on applying Bayes’ theorem with the naive belief that attributes are conditionally independent of each other. Gaussian naive Bayes supports continuous-valued features and models each as conforming to a Gaussian (normal) distribution, whereas Classical naive Bayes supports categorical features and models, each in line with a multinomial distribution.

The accuracy of the classifiers was determined by running 10 cross-fold validation and then these averages were compared directly.

### Experiment 2: Multiclass Classification With Augmented Information

#### Overview

Another approach that can be used to predict SDoHs is multiclass classification, which allows each patient to be classified under multiple SDoHs simultaneously. In addition to this mode of classification, we explored options to augment patient demographic data with personalized, publicly available information on proximity and time to travel to the nearest hospital. Thus, the goal of this second experiment was to assess the accuracy and utility of a multiclass machine learning classification with augmented personalized data for predicting patients’ SDoHs.

#### Data Set

Because the Chinatown PN study data set (experiment 1 data) was highly biased toward the language or interpreter SDoH, we decided to include data from another PN study with a different population. Thus, the data for this second experiment came from the Chinatown PN study (experiment 1 data) and the DuPage PN study, a research study evaluating PN for enhancing breast and cervical cancer screening timeliness and follow-up in DuPage County, Illinois [42,43]. Both the Chinatown PN and DuPage PN studies were implementation and dissemination studies that adapted PN protocols originating from the National PN Research Program [44,45]. For both studies, patient demographics and tracking log data sets included a patient’s sociodemographic information, including their age, income range, and education level. The data set from Chinatown is described in experiment 1. The data from the DuPage County study provided preexisting demographic data from 478 unique patients and navigation tracking (encounter) data from 435 patients collected between 2009 and 2012. Unfortunately, the DuPage data set did not include navigation encounter notes. Therefore, using topic modeling on those data was not possible.

#### Data Preparation

To use the data as one data set, it was necessary to consolidate both data sets by selecting common attributes and excluding data points that did not overlap. The DuPage data had 1 SDoH noted for each encounter along with an action to be taken by the patient (eg, an examination). We compiled all the SDoHs across encounters and assigned them to each patient. Patients without navigation encounters were excluded from this study. This process produced complete data for 400 unique patients in the DuPage study.

As previously described, the Chinatown data recorded medical history, including barriers and interventions, already listed together for individual patients (tracked by a unique Record ID), so there was no need for reformatting in that regard. However, “barrier” or “actions taken” were separated by encounter rather than listed together, such as it was in the DuPage data. To generate a similar list as DuPage, in which all barriers and interventions for a patient are immediately associated with them, the SDoH for each individual encounter was concatenated. If a patient had no navigation encounters, that record was excluded. This resulted in 274 unique patient data points for the Chinatown set.

The next task was to combine these new data sets. Both data sets did not collect the same demographic or navigational encounter information, and when they did, the data were formatted differently. Thus, we grouped the data that was common to both sets into a larger set. The codes for SDoHs did not have a 1:1 correspondence, but most were sufficiently close that we could assign correspondences between them. The DuPage data had generic codes that were easily mapped to the Chinatown data, but they also had more specific SDoH codes under each generic code. If an SDoH was not found in the Chinatown data, we used the generic code to establish correspondence to the Chinatown code.

The selection of patient demographic attributes did not have direct correspondence. For example, for employment, DuPage had only 3 categories (“unemployed,” “part-time,” or “full time”), whereas the Chinatown data broke this attribute down further differentiating unemployed from retirees, homemakers, and students. In cases in which direct conversions could not be made, values from attributes were converted to the system (DuPage or Chinatown), in which most alternative values were provided. For instance, employment status was formatted to follow Chinatown employment categories, because the latter provided more options. Furthermore, any values entered in a foreign language were converted to “none.” Any codes for values that were not specified in the DuPage codebook were changed to “chose not to answer” or “other” if these were available codes for the corresponding attribute in the Chinatown data set. Otherwise, they were changed to “none.” Numerical fields with missing values were replaced with average values for that attribute.

We grouped the 3 most frequent barriers (SDoHs) and the 3 most frequent interventions received. Selecting the most frequent barriers and interventions for a patient represented the most impactful or persistent obstacles they faced. Therefore, the model that these data would be fed into would be built to predict multiple barriers and multiple interventions per patient, up to 3. Frequencies of their appearance were counted from the consolidated lists produced earlier, and the 3 most common values were entered into the 3 new barrier fields or intervention fields.

#### Augmenting the Data

Given the tracking history and counting dates of services, a field was calculated for the number of navigation encounters associated with each patient, thus capturing the intensity of encounters. The mean (16.94) and SD (13.06) of encounter counts were determined. A new field (encounter range) was created, and patients received a value of L, M, H, or VH for low, medium, high, or very high, respectively. The upper thresholds for these categories were based on the SD, with visit counts <3 labeled as L, between 3 and 16 as M, between 16 and 29 as H, and >29 as VH.

Although patients’ specific addresses were not available, we used their zip codes and the Google Maps API to determine the patient’s nearest hospital, distance in kilometers, potential driving time, and potential travel time on public transportation in the middle of the day. Public transportation data are not always available because of the API’s own limitations; therefore, missing values were replaced with average transit time values.

Finally, all fields containing string (text)-type data were *binarized*, that is, all distinct strings were converted into columns, and each data point had a 1 or 0 for the attribute, depending on whether the text column contained that string. The classification model was required to predict 3 potential classes per patient (the top 3 SDoHs). Each SDoH, therefore, had its own column, and each patient had 1 or 0 in the top 3 SDoHs extracted as described earlier.

#### Machine Learning Model

The multilabel classification model involved a standard approach to convolutional neural network [46] using TensorFlow and Keras. The model consisted of 9 layers. The input layer was a 1D convolutional layer with a kernel width of 3, 10 filters, and a rectified linear unit activation function. The rectified linear unit function is a commonly used activation function with convolutional neural networks owing to its flexibility in approximating functions with less expensive operations than other activation functions. The input shape was the number of features per patient.

The filter and kernel values were determined after experimenting with a subset of data. A 1D MaxPooling layer was included after the kernel, with a pool size and stride value of 2. This reduces the dimensionality of the data by abstracting them, making them more general, and avoiding overfitting. The pool size and stride values were the results of experimentation to maximize accuracy.

Together with the MaxPooling layer, a convolutional layer aided in reducing the impact of smaller values in the feature set and gave them less weight in the final predictions. Subsequently, a Flatten layer was included to reduce the dimensionality, again reducing the computational cost by reducing the number of parameters to learn and avoiding overfitting. The remaining layers further abstract data features and were a series of 3 pairs of Dropout layers and Dense layers. Each Dropout layer drops a 25% proportion of the incoming values. Each Dense layer has a decreasing number of nodes, equal to a multiple of the number of classes. The first Dense layer has triple the number of output nodes, the next has twice the number of output nodes, and the final Dense layer has an equal number of nodes to classes. The output layer uses a sigmoid activation function to ensure that the value of each output node is considered independent of one another, which is necessary for multilabel classification. The model uses the binary cross-entropy loss function to determine the final class probabilities independently. Combined with the previous sigmoid activation function, this model produces the probability of each class being included in the given set of classes for a patient.

The model was evaluated through 10-fold cross-validation. K-fold cross-validation allowed repeated iterations of training and testing the model with different data splits each time.

We also explored the classification of visit intensity to determine whether demographic data could predict the intensity of visits per patient (L, M, H, and VH). Support vector machine algorithms were used for this task.

### Ethics Approval, Informed Consent, and Participation

The Northwestern University Institutional Review Board approved all study procedures with institutional review board #STU00006041 and #STU00059420. Written informed consent was obtained from all participants of the DuPage PN Collaborative and Chicago Chinatown PN Program. Participants were compensated US $50 (Chinatown study) and US $20 (DuPage study) in the form of gift cards for completing surveys. All study personnel were trained in the Collaborative Institutional Training Initiative and approved by the institutional review board. Data used in this analysis were deidentified to protect the privacy and confidentiality of the study participants.

## Results

### Results of Experiment 1

**Figure 7:**
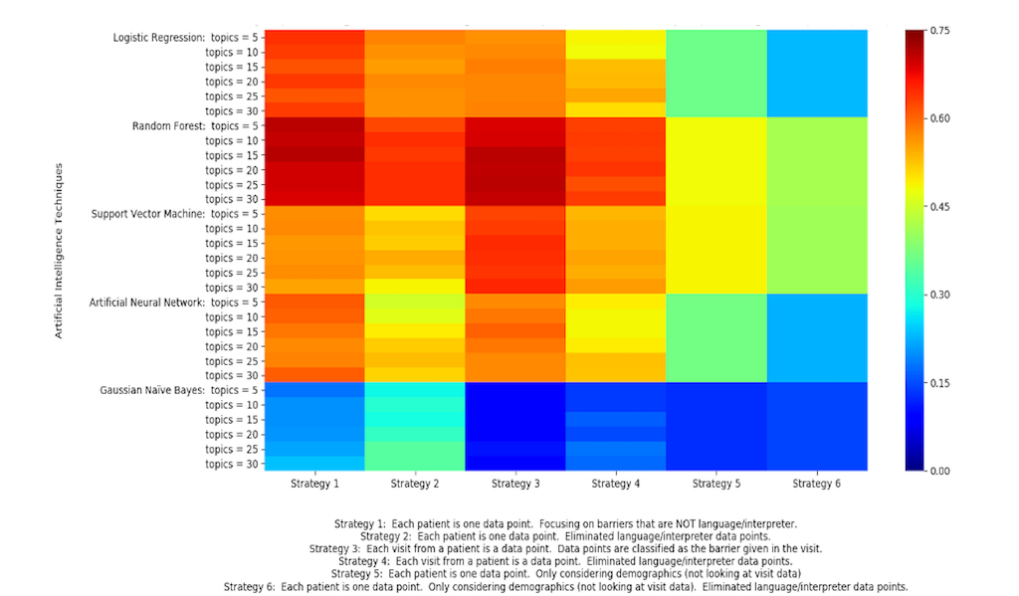
Accuracy for all tests (ranges from 0.00 to 0.75). Each rectangle represents different data preparation strategies, machine learning algorithms, and number of topics from latent Dirichlet allocation.

In the first experiment, as detailed in the *Methods* section, we compared 6 strategies and 5 learning algorithms. The heat map in Figure 7 shows the accuracy of the various machine learning algorithms over different data preparation strategies as they use different configurations of LDA attributes (number of topics). Colors closer to dark red indicate a higher accuracy. Colors closer to blue indicate a lower accuracy. LDA was not applicable to strategies 5 and 6, which only used patient demographic data; therefore, they are shown as a block.

The random forest classifier obtained the highest accuracy among the 5 classifiers tested. It outperformed all others in data preparation strategies 1 and 3 with every configuration of LDA topics. Notably, random forest with 15 LDA topics using strategy 1 yielded the highest accuracy of 71.3%. Logistic regression and support vector machine showed moderate accuracies for strategies 1 and 3, respectively. Gaussian naive Bayes was outperformed by all other algorithms under all strategies.

**Figure 8:**
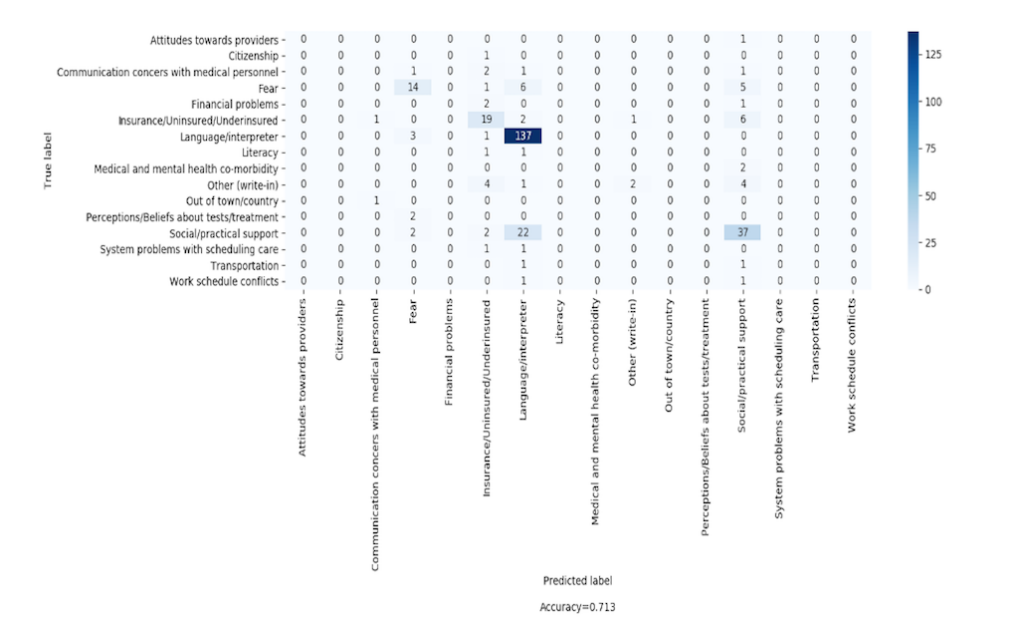
Confusion matrix of classification using random forest with data preparation strategy 1 and 15 latent Dirichlet allocation topics.

Figure 8 shows the confusion matrix for each individual SDoH for strategy 1, using random forest and 15 LDA topics—the configuration that yielded the highest accuracy. This model had an accuracy of 71.3% for predicting a new patient with a single SDoH. The confusion matrix shows the accuracy of each SDoH method. The “language/interpreter” SDoH had the best accuracy and the most instances in our data set. Moreover, for the “language/interpreter” SDoH, the actual percentage of true positives is 79.2% (137/173), whereas the false positives corresponded to “social/practical support” in 12.7% (22/173) of instances, and “fear” in 3.5% (6/173) of instances.

Other moderate rates of true positives occurred for SDoH categories “social/practical support,” “fear,” and “insurance/insured/underinsured.” Of the 22 patients classified with an SDoH of fear, 14 (64%) were accurate predictions, 3 (14%) were actually “language/interpreter,” 2 (9%) were “perceptions/beliefs about tests/treatment,” and 2 (9%) were “social/practical support.” Of the 59 patients predicted to have an SDoH of “social/practical support,” 37 (63%) were accurately labeled with the SDoH of social or practical support, whereas 6 (10%) were actually “insurance/uninsured/underinsured,” 5 (9%) were “fear,” and 4 (8%) were “other.” Of the 34 patients classified as having an SDoH of “insurance/uninsured /underinsured,” 19 (56%) were accurately classified as having an SDoH of “insurance/uninsured/underinsured,” whereas among the false positives, 4 (12%) were “other,” 2 (6%) were “communication concerns with medical personnel,” 2 (6%) were “financial problems,” and 2 (6%) were “social/practical support.”

Examining the confusion matrix for random forest with strategy 1 and 15 LDA topics (the most accurate algorithm), we can see that certain attributes can be grouped to provide a better understanding of potential SDoHs for a patient. For example, if a new patient is classified as having a “language/interpreter” SDoH, our model can predict this with 71.3% confidence. However, the classifier was 95.4% confident that the SDoH for that patient was “language/interpreter,” “social/practical support,” or “fear,” which suggests that it may be beneficial for patient navigators to pay attention to associated SDoHs when identifying a “language/interpreter” barrier.

In a similar manner, if a new patient is predicted to have (labeled with) an SDoH of “fear,” we are 96% (21/22) confident that the SDoH for that patient is “fear,” “language/interpreter,” “perceptions/beliefs about tests/treatment,” or “social/practical support.” For patients labeled as having an SDoH of “insurance/uninsured/underinsured,” there is 85% (29/34) probability that their SDoH is “insurance/uninsured /underinsured,” “other,” “communication concerns with medical personnel,” “social/practical support,” or “financial problems.” Finally, if a new patient is labeled as having an SDoH of “social/practical support,” there is an 88% (52/59) chance that the patient’s SDoH is actually “social/practical support,” “insurance/uninsured/underinsured,” “fear,” or “other.” We find that it is possible to group other attributes together and create broader classes of SDoH that are highly correlated.

However, the data are heavily biased toward the language barrier of an SDoH, which makes it difficult to extract other SDoH. In addition, although we have notes from the navigators, they did not report the SDoH in every encounter. Consequently, we were unable to determine whether the notes were meaningful for each recorded SDoH.

### Results of Experiment 2

**Figure 9:**
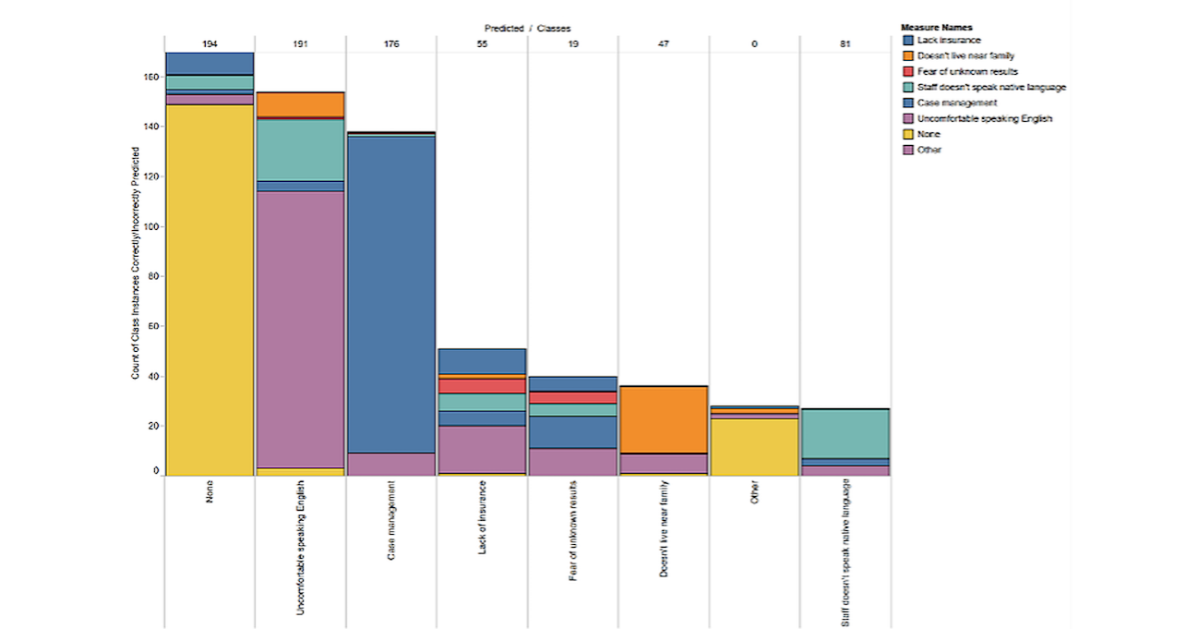
Accumulated correct predictions for each class of social determinants of health or barrier (a visualization of a confusion matrix).

In the second experiment, as detailed in the *Methods* section, we trained a convolutional neural network with augmented demographic data for each patient. Figure 9 shows the multilabel classifications for SDoH barriers and the resulting mix of correct and incorrect predictions for each class.

Figure 9 shows that the SDoH “none” was dominating the predictions, as it was the most common SDoH noted (in most encounters, patient navigators noted “none” if there were no other SDoHs besides what they had already determined, if any). Experiment 2 data show that our method is 90% accurate in detecting a language barrier and 73% accurate in predicting the need for “case management.” Other SDoHs were not predicted with any consistent accuracy.

Regarding the correlation between each SDoH and encounter intensity, the highest correlation was with “other primary language” (*r*=0.55) followed by “Spanish primary language” (*r*=0.27). When we also looked at correlations between other attributes and SDoHs, we found a positive correlation between “no near family support” and “China” being a country of origin (*r*=0.44), and a negative correlation between “no near family support” and “Spanish” being a primary language (*r*=−0.26).

These predictions were not very different from those obtained in the first experiment, and this may be, in part, because of the dominance of language as a barrier, as both data sets included mostly individuals for whom English was not their primary language. It is worth noting that in the experiment 2 data, many annotations for SDoH had the word “none” in them, despite an SDoH being recorded either at a later or earlier time point. Moreover, we were not able to fully personalize the augmented data because of the absence of specific patient addresses, but we did show a proof of concept that it is possible to augment the data with personalized information through other methods.

## Discussion

### Principal Findings

In this case study, we report on 2 experiments exploring the feasibility of using machine learning to predict the SDoHs for PN research. This study offers novel approaches to address several research gaps in the literature, including using textual data, augmenting data with personalized information, and using multiclass predictions. The findings of this study were mixed. In the first experiment, we used data from patients living in Chinatown in Chicago. We compared 5 algorithms with each other, and examined whether the text in the notes of the navigator can make a difference in the prediction of SDoHs. In this experiment, the random forest classifier with 15 LDA topics using data preparation strategy 1 (each patient is a data point) yielded the highest prediction accuracy of 71.3%. Moreover, we were able to group certain attributes to provide a better understanding of the potential clustering of SDoHs for a patient. In the second experiment, using data from both Chinatown and DuPage County, we augmented demographic information and used multiclass classification to predict SDoHs. We were able to effectively predict a handful of SDoHs but did not result in better predictions than the most accurate models in the first experiment.

Although the predictive power of our approach was limited to a handful of SDoH-related barriers and we found a few useful correlations among SDoHs, our study produced other insights. In applying machine learning algorithms to the Chicago Chinatown and DuPage PN data sets, our case study yielded some valuable lessons learned that can inform future use of machine learning for PN programs and other initiatives addressing SDoH barriers to care. As discussed, the lessons learned include (1) planning for the standardization of data sources and measurement that purposefully lend themselves to predictive analysis; (2) identifying and anticipating the intersectionality and clustering of SDoHs; and (3) being aware of model limitations.

### Standardization of Data Sources and Measurement

If we call the action of observing a patient and their associated data “x,” then a patient navigator will try to take “x” and predict a set of SDoHs “s.” Machine learning algorithms try to replicate this human prediction process by finding a function that statistically fits a large number of human predictions so that when given a particular “x” it can determine “s” as accurately as possible (f(x)=s). Because this fit is statistical in nature, it requires data that are relevant to the attribute being predicted (≥1 SDoHs). Because it is generally the case that the more data available for training an algorithm, the better the predictive results, larger data sets and replication are needed. However, a larger data set does not guarantee an accurate prediction of SDoHs. Because many studies on SDoHs target specific populations, it is not unusual to observe bias in the data [38]. For example, when working with English-learner immigrants of low socioeconomic status, it is reasonable to expect *finances* and *language* among the SDoHs. In this case, the machine learning predictor will be very accurate for these 2 SDoHs; however, it could be less useful for real-life applications, as most patient navigators will be able to notice these 2 SDoHs in their patient encounters. Accurate prediction of less-common SDoHs may be a more useful application of these algorithms, but biases in the data make these predictions hard.

Therefore, in our case study we conducted extensive data preparation for our 2 experiments, and we tried to exclude “language” as it was a very pervasive SDoH (experiment 1). Our results align with those of previous research [39]; however, we provide an alternative method to augment traditional models with textual information and individualized information from publicly available data (eg, Google Maps API).

To optimize the use of machine learning for predicting and addressing SDoHs in PN contexts, data collection protocols and data structures should be intentionally collected for machine learning purposes. First, collecting more samples with less-common SDoHs is key. It is not sufficient to collect data from a large pool of individuals, but the variety of SDoHs is fundamental for accurate statistical predictions. Second, there must be planning for multiclass prediction tasks from the outset. This includes a system for accurately recording SDoHs during encounters, a protocol to handle when new SDoHs are recorded, and when wildcard SDoHs are recorded. In experiment 2, “none” was a wildcard SDoH and, as such, very pervasive as well. Third, there should be considerations for recording additional information that helps augment the data with readily available personalized information from the web. For example, patient voice and textual data are underused, but hold tremendous potential as a source for SDoH prediction. Responses to general questions such as “How are you feeling today?” could add to the data on patient barriers, from which speech recognition can be used to identify SDoH-related topics and conversations that emerge. Automated tools with speech recognition could be used when possible; however, special care must be taken by testing them before their use, as the collection method or other external circumstances may result in the collection of unusable data. For example, if the interviews are conducted over the phone, then the audio quality is vital. This quality can be affected by interference in the call, a speech impediment, low volume, or slurred speech in older individuals. In addition, the language spoken by the patients can be a dialect. In our Chinatown data, patients do not speak Mandarin or Cantonese, but Toishanese, for which we could not find satisfactory speech recognition software. Finally, additional questions, such as “At what time are you available to go to the doctor?” together with the patient’s address and the provider’s address can provide augmented data that indicate the driving time or public transportation time it would take the individual to see a health care provider.

### Identifying and Anticipating SDoH Intersectionality

Using the random forest algorithm with data preparation strategy 1 and 15 LDA topics (the most accurate algorithm in the first experiment), we grouped certain attributes to provide a better understanding of the potential correlations of SDoHs for a patient. These findings underscore the growing recognition that SDoHs are not discrete phenomena; they operate in complex, integrated ways. Their intersectionality should be anticipated and identified for machine learning to help advance the work of patient navigators. The National Institute on Minority Health and Health Disparities has proposed a multidimensional research framework to understand and address minority health and health disparities [47]. This framework conceptualizes SDoHs as involving a wide array of health determinants spanning different domains of influence and multiple levels of influence within each domain. As the National Institute on Minority Health and Health Disparities research framework indicates, there are not just single social factors but rather their interrelatedness may have an overall effect on an individual’s health outcomes. Moreover, as the framework suggests, a combination of several determinants could play a larger role in a person’s health than any single determinant. Building a predictive model that considers this framework as part of its predictive algorithm could be a powerful tool for patient navigators and health care providers. It would allow them to recognize and address a patient’s unique set of social determinants to improve health outcomes for that individual. In the future, methods such as Conditional Random Fields could not only be used to predict multi-SDoH outcomes but also the strength of the correlation among them for every single patient. Methods such as convolutional neural networks [46] can leverage these correlations for more accurate outcomes.

### Limitations

The limitations of this research can be grouped into 2 main areas: limitations in the models and limitations in the experiments. Regarding limitations of the models, multiple recent reports and systematic reviews have brought attention to issues of bias in health models of machine learning and the unintended harms that can arise when using machine learning for prediction [48-51]. In the case of Chinatown and DuPage PN data, our patient population consisted primarily of low-income individuals whose primary language was not English; thus, the model classifications were biased toward language and financial (eg, health insurance) SDoH barriers. If used for prediction purposes, machine learning algorithms would primarily guide navigators toward focusing on patients’ language and health insurance–related barriers while largely ignoring other SDoH barriers. This is not to say that other SDoH barriers do not exist in these patient populations, but because of the bias in the data set from which the algorithms were trained, 1 unintended harm that may arise is that other SDoHs are at risk of being ignored. Although the findings and lessons learned from this study demonstrate the promising use of machine learning algorithms in predicting SDoHs in PN work, there are some considerations that support the retention of human experts (eg, patient navigators) in many aspects of identifying and addressing SDoHs. As noted earlier, the predictions made by machine learning algorithms are only as good as the data available for training the algorithms. Thus, without better data, navigators will still need to rely on manual identification of SDoHs to ensure that less-common but pertinent SDoHs are addressed to truly provide patient-centered care.

In terms of methods, a limitation of the 2 experiments was the small sample size. As discussed earlier, small and biased samples are detrimental to the detection of marginal patterns. In addition, because of its small size, we did not use validation data sets, but larger training and testing data sets. Another limitation is that these experiments used data collected from PN research studies. Data from other real-world PN contexts may vary in quality and content; therefore, further investigation is required.

### Future Work and Conclusions

Although our focus was on predicting SDoHs, we envision that machine learning can have a broad range of applications in the field of PN, tailoring intervention delivery (eg, supporting PN decision-making), informing resource allocation for measurement, and augmenting PN supervision. Recent findings from intervention research studies in the health care space also support the potential use of machine learning to enhance interventions. For example, Pfob et al [52] tested 3 machine learning algorithms to predict outcomes at a 1-year follow-up to facilitate patient-centered decision-making in women with breast cancer. In another example, O’Donovan et al [53] developed an open-access machine learning web application (CHWsupervisor) to support community health worker supervision in Uganda and Kenya. They found that CHWsupervisor had “moderate” predictive accuracy compared with human coders in coding instant messages exchanged between community health workers and their supervisors and noted that machine learning approaches hold promise, but that supportive supervision still requires a level of human expertise because of the complexity of exchanges that often require nuanced interpretation [53]. In this study, we similarly experienced that preparing data for machine learning was challenging because of the nuanced interpretation required to convert the navigator’s comments on each visit (text) into meaningful numerical data.

However, any application of machine learning to PN work will be constrained by the availability of data from which to train algorithms. Thus, attention is needed on bolstering data that can be made useful for machine learning algorithms. Recent efforts to link SDoH-screening responses from electronic health records with existing medical coding tools may be vital to this effort, as exemplified by the National Association of Community Health Centers’ preliminary linkages between its SDoH screening items and the International Classification of Diseases, Tenth Revision codes [24]. Machine learning has previously been used for thematic analysis of qualitative data [39]; therefore, speech recognition can potentially be used on patient voice and text data to form another rich source of data for machine learning algorithms.

Finally, the sheer amount of data is not necessarily sufficient. A variety of SDoHs is essential for a bias-free data set. Sometimes, this may not be possible, and in that case, researchers will need to focus on statistical methods that simulate instances of patients with a diverse pool of SDoHs based on existing data or boost classification with a combination of methods and voting strategies [54,55].

Despite these limitations, this case study illuminates the value of machine learning in offering new opportunities to predict SDoHs to enhance the effectiveness of PN in improving health outcomes in diverse patient populations, and further investigation is warranted. Future work should involve a more intentional data collection process, together with larger data sets that allow our models to make better inferences and allow researchers to run validation data sets for better classification outcomes.

## Abbreviations

API: application programming interface
LDA: latent Dirichlet allocation
PN: patient navigation
SDoH: social determinant of health

## Data Availability

The data sets used and analyzed during this study are available from the corresponding author upon request.
